# Supplementary figures and images for: How Automated Techniques Ease Functional Assessment of the Fetal Heart: Applicability of MPI+™ for Direct Quantification of the Modified Myocardial Performance Index
Source: Diagnostics (Basel). 2023 May 11;13(10):1705. doi: 10.3390/diagnostics13101705 (PMC10217300; doi:10.3390/diagnostics13101705)

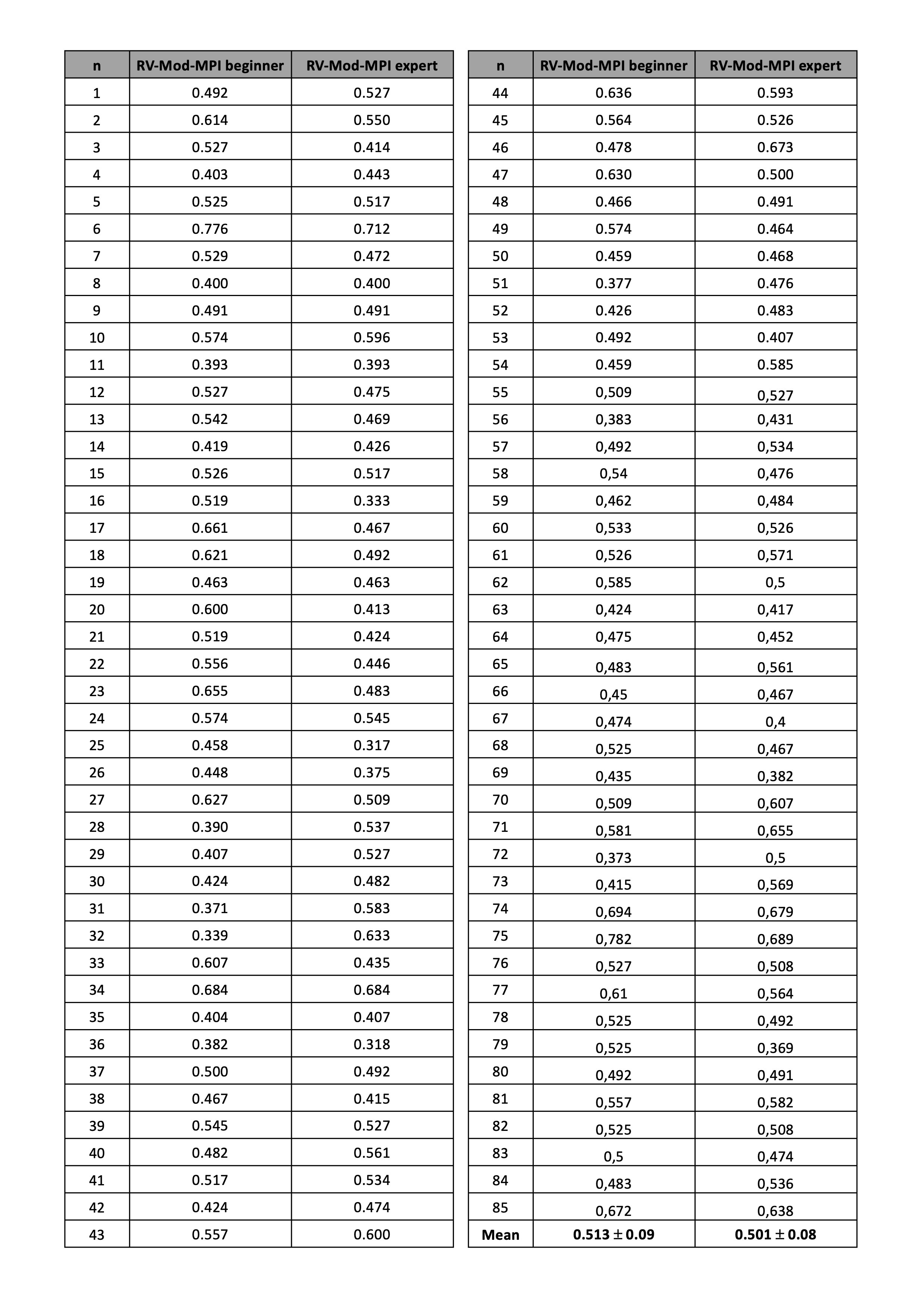

Supplement: Supplementary file 1 [file diagnostics-13-01705-s001.zip › Table S1 - MPI-Values beginner and expert.tiff]
